# Enlightening activation gating in P2X receptors

**DOI:** 10.1007/s11302-022-09850-w

**Published:** 2022-02-21

**Authors:** Christian Sattler, Klaus Benndorf

**Affiliations:** Institut Für Physiologie II, Universitätsklinikum Jena, Friedrich-Schiller-Universität Jena, 07740 Jena, Germany

**Keywords:** P2X receptors, Activation gating, Voltage-clamp fluorometry, Patch-clamp fluorometry, Ligand binding, Computational modeling

## Abstract

P2X receptors are trimeric nonselective cation channels gated by ATP. They assemble from seven distinct subunit isoforms as either homo- or heteromeric complexes and contain three extracellularly located binding sites for ATP. P2X receptors are expressed in nearly all tissues and are there involved in physiological processes like synaptic transmission, pain, and inflammation. Thus, they are a challenging pharmacological target. The determination of crystal and cryo-EM structures of several isoforms in the last decade in closed, open, and desensitized states has provided a firm basis for interpreting the huge amount of functional and biochemical data. Electrophysiological characterization in conjugation with optical approaches has generated significant insights into structure–function relationships of P2X receptors. This review focuses on novel optical and related approaches to better understand the conformational changes underlying the activation of these receptors.

## P2X family — overview

**Fig. 1:**
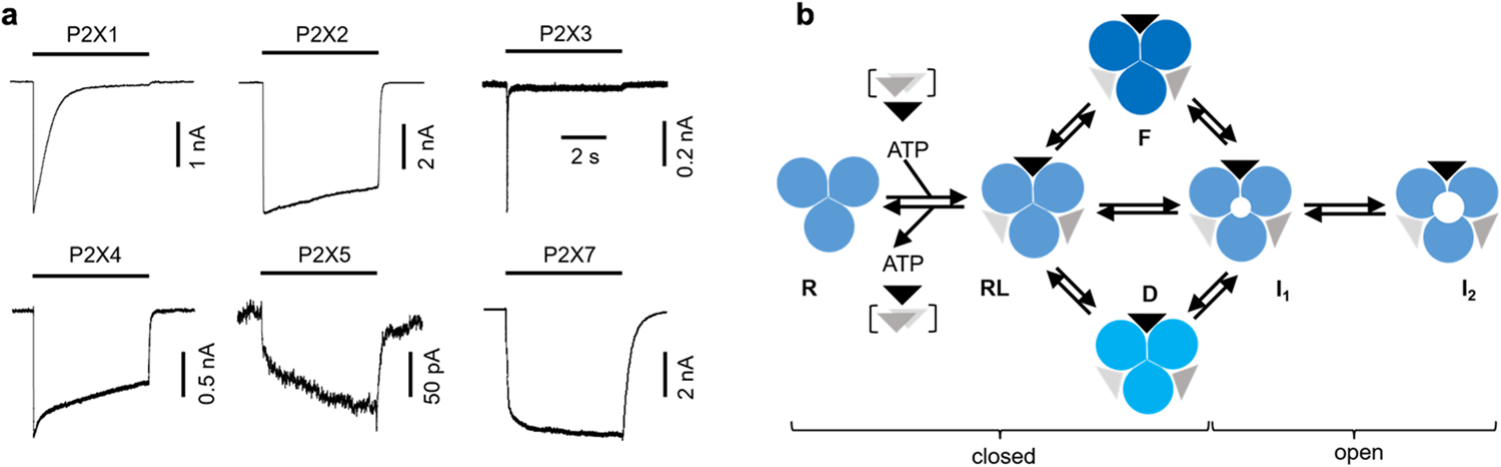
Functional properties of P2X receptor types. **a** Representative examples from own recordings from rat P2X receptors at saturating ATP concentration (100 µM for P2X1-5 and 1 mM for P2X7) expressed from HEK cells in the whole cell configuration. Recordings were performed with buffered and divalent free symmetrical sodium chloride solutions (142 mM) at a holding potential of − 50 mV. **b** Simplified kinetic model for P2X receptors. A receptor is built by three subunits around a central pore. Upon binding of one to three ATP molecules, the resting state (*R*) forms the receptor ligand complex RL. Depending on the isoform, different isomerizations are possible, including a closed flipped state *F*, a closed desensitized state *D*, as well as the open states *I*_1_ and *I*_2_

ATP is not only an energy source; it is also a signaling molecule and, thus, a player in the purinergic system [1, 2]. Together with other purine and pyrimidine nucleotides, it can stimulate two classes of receptors, metabotropic P2Y receptors and ionotropic P2X receptors (P2XRs) [3]. Mammals express seven subunit isoforms (P2X1-P2X7) that are ubiquitary expressed. Their function is highly diverse, ranging from synaptic transmission and processing of pain and inflammation to the regulation of blood pressure [4]. Therefore, these receptors raised significant attention as potential drug targets [5]. Except for P2X6, all subunit isoforms assemble to functional homotrimers [6]. P2X1Rs and P2X3Rs are the isoforms with the highest apparent affinity. They are activated at ATP concentrations below 1 µM [7, 8]. Unique for P2X7R is that, in comparison to the other P2XRs, their apparent affinity is lower by two orders of magnitude [9]. The receptor kinetics are also diverse and isoform-specific: activation of P2X1-4Rs is fast compared to P2X5Rs and P2X7Rs whereas desensitization is fast in P2X1Rs and P2X3Rs whereas it is slow in P2X7Rs (Fig. 1a) [10]

For the description of the diverse agonist responses, isoform-specific distinct states have been proposed (Fig. 1b). The closed resting state *R* can bind up to three ATP molecules, forming the receptor-ligand complex RL. This complex can transit to the open state *I*_1_. After the prolonged presence of ATP, a second open state *I*_2_ with reduced selectivity has been postulated by several studies for P2X2Rs, P2X4Rs, and P2X7Rs [9, 11–13], but its existence is still a matter of debate [14–18]. This postulate is supported by the observation that big organic molecules like NMDG, ethidiumbromide, or YO-PRO-1 can pass the membrane upon ATP stimulation, even in purified and reconstituted panda P2X7Rs [9, 19]. Major support for the existence of the megapore state *I*_2_ arises from shifts of the reversal potential, thereby assuming a stable ion concentration on both sides of the membrane. In contrast, the work by Li and coworkers [15] has shown for P2X2Rs that this assumption does not hold true. This study doubts the interpretation of the observed reversal potential shift. Furthermore, the distinct desensitization for P2X1Rs and P2X3Rs requires another ligand-bound closed state D, which is formed either after or in parallel to the ligand-induced opening and characterized by an affinity for ATP in the nanomolar range [20–22]. For P2X2Rs, an intermediate flipped state *F* of the closed channel has been proposed [23–25].

The formation of heterotrimeric receptors has enabled nature to enhance the functional diversity of P2XRs. So far, at least eight heteromeric channels have been identified on functional grounds, mainly by co-expression in recombinant systems and by current characteristics differing from those of the homotrimers, sometimes accompanied by immunoprecipitation assays or, exceptionally, by atomic force microscopy. These heteromers are P2X1/2R [26, 27], P2X1/4R [28], P2X1/5R [29–31], P2X2/3R [8, 32, 33], P2X2/5 [34], P2X2/6R [35, 36], P2X4/6R [37], and P2X4/7R [38].

## Structure–function relationships in P2X receptors

**Fig. 2:**
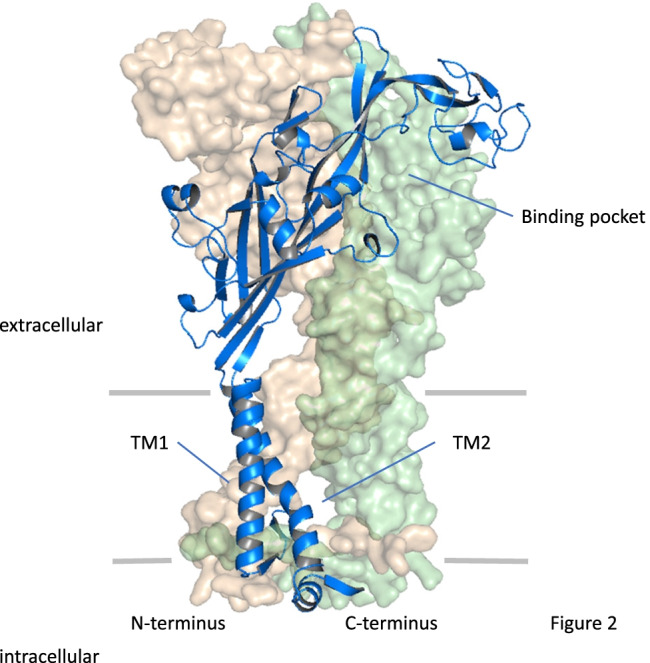
Trimeric rat P2X2 receptor as representative example. One subunit is shown in ribbons whereas the other two subunits are shown in surface representation. The rat P2X2 structure (UniProt accession ID: P49653) was generated by homology modeling using the human P2X3 channel in the ATP-bound open state (PDB: 5SVK) as a template with the SWISS-MODEL server. Modified figure from [119]

To date, 27 high-resolution structures have been resolved for P2XRs [5], and the number seems to grow rapidly. The starting points were the crystallization of the P2X4R from zebrafish (zfP2X4) in the closed and ATP bound open state [39, 40], confirming the trimeric nature of P2XRs, as postulated earlier by biochemical cross-linking experiments [41], disulfide bond formation between subunits [42], and single-channel recordings together with mathematical modeling [43]. Moreover, the structures revealed a dolphin-like shape of the subunits (Fig. 2). The dolphin tail is built by the pore-forming transmembrane domain 2 (TM2) and the pore-assisting transmembrane domain 1 (TM1). The body is organized in the large ectodomain by β-sheet structures, forming lateral fenestration sites for ion access. Flexible domains branch from the body, including head, dorsal fin, and right and left flipper. The binding pocket for ATP is positioned between two subunits, formed by the head and left flipper of one subunit and the dorsal fin of the adjacent subunit. The P2X3R was the first crystallized receptor containing relevant intracellular domains, and it was crystallized in a variety of states, including the resting, agonist-bound open, closed, and desensitized, as well as antagonist-bound closed states [44]. Herein, a functionally relevant cytoplasmatic cap was identified, that stabilizes the open conformation and generates lateral fenestrations for ion egress. In 2019, the full-length structure of the P2X7R was obtained [45]. Characteristic for this subtype is the long cytoplasmatic C-terminus harboring binding cites for proteins, zinc ions, and guanosine nucleotides. Furthermore, it contains palmitoylated cysteine residues forming an anchor of the pore-lining helix to the membrane, preventing the receptor from desensitization.

As outlined above, the three ATP-binding sites of a P2X receptor are located at the three subunit interfaces. By site-directed mutagenesis and electrophysiology, a bunch of amino acids were identified to contribute to ATP binding, encompassing in the P2X4R of the zebrafish the basic residues (K69, K71, R290, K308), aromatic (F183, F289), and polar residues (T184, N288). Exploring further details involved in the binding of ATP to several subtypes, the reader is guided to excellent reviews by Chataigneau and Hausmann [46, 47]. The involvement of multiple residues in ligand binding by evolution has caused that the natural ligand ATP is bound to P2XRs with the known high apparent affinity and specificity [48], though numerous derivatives of ATP and related compounds with either elevated or reduced apparent affinity have been created [49], suggesting that essential aspects of the binding process are increasingly understood. It should be mentioned that, strictly speaking, it is two negatively charged forms of ATP that bind to the binding sites, ATP^4−^ and MgATP^2−^, and that the binding of these charged ATP forms is even subunit-specific [10]. These aspects are not considered in the present review.

## Definition of functional parameters

In an attempt to be in the terminology of functional parameters as clear as possible, we first specify our terminology for the example of a homomeric receptor containing a number of identical binding sites.

### Ligand-receptor interaction at equilibrium

At a constant ligand concentration in the environment of a receptor, an equilibrium between bound and unbound receptors is formed.

#### True affinity

The true affinity is defined by the equilibrium association constant, ***K***_**A**_, or its reciprocal, the dissociation constant, ***K***_**D**_. It is highly remarkable that at unchanged true affinity subsequent conformational changes of the receptor can significantly alter ligand binding [50]. Ligand binding can be measured directly by ligands tagged e.g., by radioactive or fluorescence labels. Hence, direct measurement of the concentration of half-maximum binding, *BC*_50_, does not report the true affinity but the occupancy of a ligand at the binding site. The true affinity itself can only be determined if including subsequent conformational changes and fitting respective schemes.

#### Downstream responses

Instead, ligand binding to a receptor is regularly quantified by functional assays, determining a downstream response of the receptors when applying a ligand. Depending on the assay, a downstream response can be of a highly different nature, ranging from conformational changes in the receptor itself, as e.g., current amplitudes for receptor channels, to any other biological responses, often far more downstream with respect to ligand binding. These downstream responses can have three relevant aspects:

1. The first aspect is the concentration generating the half-maximum response. It is termed herein apparent affinity. The term “apparent” indicates the concentration generating the half-maximum response. The apparent affinity can differ significantly from the true affinity [50]. For an agonistic ligand, as ATP for P2X receptors, the apparent affinity is usually quantified by the concentration of half-maximum activation, ***EC***_50_. In pharmacology, the apparent affinity is equivalent to the term potency.
2. The second aspect is the slope of the normalized concentration-activation relationship, providing a rough measure for cooperativity of the subunits in the activation process. The slope is usually quantified by the Hill coefficient, as obtained by fitting the normalized concentration-activation relationship with the Hill equation. A value exceeding 1, 2, 3…indicates the involvement of more than 1, 2, 3… active binding sites, respectively. However, despite its wide use, the Hill coefficient assumes infinite cooperativity and is therefore physically nonsensical.
3. The third aspect is the efficacy, ***E***, i.e., the amplitude of an effect at saturating ligand concentration with respect to the maximum possible effect, *E*_max_, at a saturating ligand concentration of a full agonist. If *E* = *E*_max_, a ligand is termed a full agonist whereas, if *E* < *E*_max_, it is termed a partial agonist.

### Ligand-receptor interaction distant from the equilibrium

In addition to these equilibrium parameters, jumps of the ligand concentration can provide additional information about the operation of a receptor. This allows to determine, e.g., so-called on- and off-rates when applying and washing a ligand, respectively, and when employing kinetic models for interpretation transition rates between states.

## Functional properties of the pore

Before coming to the focus of this review, the gating of P2X receptors, a few functional aspects of the P2XR pore, are considered.

### Unitary conductance

As shown above, all P2X receptors apart from P2X6 receptors, which do not oligomerize [51], form functional homotrimeric channels, i.e., each subunit contributes to the pore. Compared to other ligand-gated ion channels, the single-channel performance of P2X receptors is characterized by a marked flicker of the unitary currents, i.e., the conductance, making all respective data to some extent vague. The estimated values are in the range of other ligand-gated ions channels: ~ 12 pS for P2X1R [52], ~ 21–35 pS for P2X2R [43], ~ 6.6 pS for P2X3R [53], ~ 9–18 pS for P2X4R [52, 54]; < 5 pS for P2X5R [55], and 9–13 pS for P2X7R [56]. So far, there is no systematic approach that allows to understand how the different subunits in heteromeric P2X receptors define the unitary conductance.

### Residues specifying the conductance

Myriads of data have been gained on residues determining the unitary conductance. In contrast to many other channels, the present view is that the conductance is determined not exclusively by residues of the TM2 helix, lining the pore, because relevant sites co-determining the unitary conductance were also identified in the lateral extracellular channel portals. This unusual complexity is possibly the reason why the conductance is less well fixed and can adopt many open states, generating the mentioned flickery performance of the open channel [43, 52, 57].

### Selectivity and megapore

Primarily, P2X receptors are categorized as non-selective cation channels because the physiologically present Na^+^ and K^+^ ions permeate similarly well, and, as typical for other non-selective cation channels, also the other alkali ions Li^+^, Rb^+^, and Cs^+^ permeate the channels, among which Li^+^ ions permeate best [52, 58–60]. In addition to these main chemical group 1 ions, all P2X receptors are permeable for Ca^2+^ ions [61] generating under physiological conditions partial Ca^2+^ fluxes of 5 to 16% [62] which is exceptionally relevant for function because they trigger diverse signaling cascades. Wild-type receptors prefer cations over anions by a factor > 10. Exceptions are P2X5Rs which in some species show significant Cl^−^ permeability. For a comprehensive review on ion selectivity, the reader is directed to Samways et al. [62]. With respect to other channels, a further highly unusual feature of P2X7 receptors is that, upon sustained exposure to an agonistic ligand, they can substantially widen their pore, forming in a slow process a so-called megapore [13]. The megapore allows for permeation of much larger molecules than ions, as for instance NMDG^+^ (*N*-methyl-d-glucamine) [63] or the cationic dye ethidium [64, 65]. Also, P2X2R and P2X4R can substantially widen their pore. Single-channel recordings of P2X2 receptors suggested that organic molecules can pass the pore immediately after the ATP exposure [66]. It was also shown for human P2X7Rs in single-channel recordings that these big organic molecules can pass the pore [56]. Moreover, the postulated change in the selectivity parallel to receptor facilitation observed in whole-cell recordings [13] is in contrast to an unchanged agonist-opened selectivity filter in TM2 observed in single-channel recordings [67]. Even after decades of intensive research, the nature of the megapore is a matter of debate.

Since we will focus here on novel approaches for analyzing the gating, the reader is directed to a series of excellent reviews on P2X channels, including extended information about conductance, selectivity, and megapore formation [14, 48, 49, 59, 62]

## Activation gating

The intramolecular processes of the activation gating in P2X channels are presently also only poorly understood, i.e., how the ATP ligand binding to the three available binding sites is translated by a channel to the gate, opening the pore. Though there are three binding sites, it is even controversial whether or not partial liganding evokes partial activation and whether full activation requires the occupancy of all three binding sites. Regarding the number of the subunits involved in activation, three previous studies proposed that only two activated subunits are required for channel activation [68–70] whereas other studies provide evidence that all three subunits are involved [42, 43, 68, 71]. Moreover, it remains finally unanswered, whether one liganded subunit can activate the channel at all, and if yes, to what extent. Since the ATP binding takes place distant from the membrane plane, the signal elicited by ligand binding must propagate to the transmembrane gate along the protein. Here, a central role of the β-14 sheet has been identified [69]. This structure connects within a subunit the ATP binding site with the transmembrane pore-forming TM2 helix. In this process, the β-14 sheet interacts with the β-1 sheet of the adjacent subunit [72].

There is experimental evidence from P2X2 receptors that each ATP-binding signal propagates first along the own subunit and only then spreads equally to all three subunits towards the pore [69]. As a consequence, there is a closed-open transition to which each of the three subunits contributes [73]. In another study, five distinguishable steps were identified in activation, including ATP binding, tightening of the binding “jaw,” flexing of the lower body regions, expansion of the lateral fenestrations, and subsequent pore opening [74].

Hence, despite knowing the channel structure of several isoforms (see above) in both the closed and the open conformation, there is still a big gap of knowledge between the statics of the structures and the dynamics of the molecular mechanics upon activation. Therefore, new functional approaches are required to provide new insights feasible to fill this gap.

## Methods to analyze activation gating preceding pore opening

### Measuring conformational changes

**Fig. 3:**
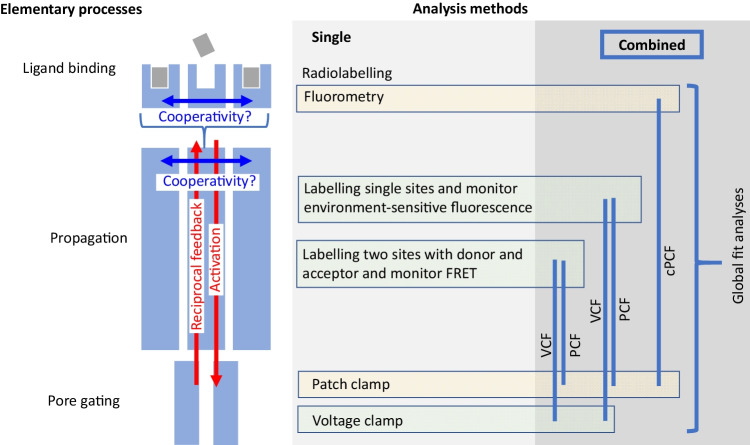
Methodological approaches to investigate elementary processes in receptors as ligand binding, propagation of the signal within the receptor, and pore gating. Electrophysiological measurements can be combined with optical readouts as used in voltage-clamp fluorometry VCF, patch-clamp fluorometry PCF, or confocal patch-clamp fluorometry cPCF. Global fits are powerful tools to specify schemes for binding and gating steps

Fluorescence labeling of proteins by either fluorescent proteins or dyes linked to defined amino acids is widespread tools in biomedical research. The most frequent aims of such studies are to localize the desired proteins in cells, tissues, and organs as well as to control expression in heterologous expression systems. Other studies use the labeling of proteins to report functional parameters of the proteins. We will focus here on understanding the processes underlying the propagation of the binding signal to the pore by conformational changes within the channel protein (Fig. 3), and methods to investigate them. There are two major experimental lines: (1) to label one site in the receptor with an environment-sensitive dye and read out changes in fluorescence intensity generated by quenching when activating or modulating the receptor and (2) to label two sites in the protein, one by a fluorescence donor and the other by a fluorescent acceptor, and to quantify the so-called Förster resonance energy transfer (FRET) [75, 76]. Before summarizing recent developments on P2X receptors, we will shortly consider some studies on other receptors and expression systems.

There are two categories of expression systems: *Xenopus* oocytes and mammalian or other cell lines with much smaller cells. Due to the strong differences in cell size, there are also two different experimental approaches to study the context between activation gating and conformational changes. The first approach is to voltage-clamp whole *Xenopus* oocytes, performed either with two-microelectrodes or in cut-open chambers. This widespread method is a powerful technique to measure ion currents through a very large number of heterologously expressed channels. If combining this technique with optical measurements of fluorescence signals from fluorophores, reporting about conformational changes, two orthogonal signals can be recorded in parallel. This technique, called voltage-clamp fluorometry (VCF), was originally introduced in 1996 by Mannuzzu and coworkers [77]) and has been applied since then to analyze numerous other channels.

The second approach is based on the patch-clamp technique which allows to measure currents in the much smaller cells, but also in patches of membranes, which can be cell-attached or excised from the cells [78]. Patches can also be obtained from *Xenopus* oocytes. Also, the patch-clamp technique can be combined with optical measurements to monitor in parallel highly diverse signals, depending on the fluorophores put into the proteins under study. This technique, termed patch-clamp fluorometry (PCF) has been introduced by Zheng and Zagotta in 2000 [79].

Both methods are useful to study the function of the labeled receptor or channel proteins in relation to states and time courses of activation and deactivation.

#### Signals generated by quenching fluorophores

##### General aspects

The method of site-directed fluorescence was first used to analyze the gating apparatus of the voltage-dependent Shaker (IR) K^+^ channel [77]. As label, the authors used tetramethylrhodamine maleimide (TMRM), reacting with cysteine positioned in the channel. Because TMRM changes its photophysical properties depending on the environment, these quenching signals can be used to study local conformational changes around the TMRM probe at high time resolution. The outcome was that the labeling depends on the position and the membrane potential. The fluorescence signals matched with the moved gating charge. In a subsequent study, also specific differences between the moved gating charges and the fluorescence signals were identified which could be mechanistically interpreted [80, 81]. Since then, the method of site-directed fluorescence quenching has become a powerful tool to analyze conformational changes also in numerous ligand-gated receptors and channels, for example, for reporting molecular rearrangements in nAchRs [82], GABA-ARs [83], spHCN pacemaker channels of the sea urchin [84], or cys-loop receptors [85].

Two examples of respective studies with the PCF approach are parallel measurements of the channel function and structure in inside-out patches with fluorescence spectroscopy, thereby evaluating the quenching by tryptophan [79, 86]. The method is clearly less frequently used, presumably because the optical signals are much smaller. However, the possibility to work in excised patches provides several advantages, among them to get rid of disturbing intracellular structures and gaining the possibility of very rapid solution jumps. A variant of the method, confocal patch-clamp fluorometry (cPCF), will be discussed below.

##### P2X receptors

**Fig. 4:**
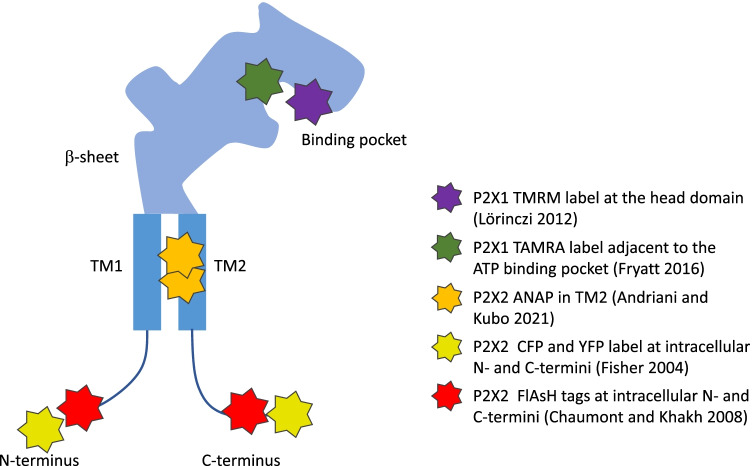
Different strategies to label P2X receptor subunits for monitoring conformational changes are illustrated in a cartoon of a single subunit, thereby ignoring that a true binding site is formed by two subunits. Rhodamine derivatives TMRM and TAMRA were introduced on mutated cysteine residues near the ATP binding pocket. Voltage-dependent movement was monitored by ANAP incorporation in the TM2 domain. Cyan and yellow fluorescent proteins or FlAsH tags at the cytosolic termini report conformational changes associated with changes of the ion selectivity

Since its introduction, it took 12 years until the first VCF study on P2XRs came up. Using again TMRM as label, Lörinczi and coworkers determined amino acids in the rapidly desensitizing P2X1R that monitor conformational changes associated with the ligand binding (Fig. 4) [87]. The authors showed that the cys-rich head domain undergoes substantial movement during P2X1R opening and desensitization as well as that the substituents at the ribose moiety of the ATP analogous ligands Bz-ATP and TNP-ATP are specifically detected by TMRM.

VCF was also used in the same P2X isoform to record in parallel ion channel activity and conformational changes at several positions of the extracellular loop of the receptor during not only agonist binding and desensitization but also during recovery from desensitization [88]. The authors showed that ATP evokes distinct conformational changes adjacent to the agonist-binding pocket that are related to channel activation and desensitization. The authors suggested that the intracellular portion of the receptor can regulate the recovery from desensitization. This showed for the first time that this is by a mechanism independent of changes in the extracellular domain, suggesting the existence of a distinct desensitization gate. In a successor study on the same receptor, the authors analyzed with VCF the K190C mutant, that is adjacent to the ligand-binding pocket [71]. They labeled the cystein with the fluorophore MTS-TAMRA and monitored changes in fluorescence when applying an agonist. In combination with molecular dynamic simulations, they attributed the observed fluorescence time courses to conformational changes. Moreover, the data led the authors to the conclusion that P2X1Rs are normally activated by three ATP molecules, matching earlier results of Ding and Sachs JGP 1999. VCF was also used to study with the help of the fluorescent non-canonical amino acid ANAP structural rearrangements of rat P2X2Rs as function of membrane voltage, elaborating specific interactions of amino acids in the TM1 and TM2 domain [89].

In another approach on P2X2Rs, labeling the N- and the C-terminus with FlAsH to specific 4C tags combined optical and patch-clamp recordings in the whole-cell configuration enabled to resolve the kinetics of slow conformational changes associated with the *I*_2_ state [90]. Together, though the number of studies on P2X receptors combining electrophysiological and optical recordings is still rare, the potential of this approach and PCF is high to provide further relevant contributions to our understanding of these relevant receptors.

#### Signals generated by FRET

##### General aspects

Förster resonance energy transfer (FRET) is a process that causes the transfer of energy from one light-sensitive molecule (donor) to another light-sensitive molecule (acceptor) by a non-radiative dipole–dipole coupling [91]. Notably, the efficiency of this energy transfer is inversely proportional to *r*^6^ where *r* is the distance between donor and acceptor. This dependency makes FRET usable as a highly sensitive molecular ruler, in particular at conformational changes (for reviews see e.g. [92, 93]).

It is therefore not surprising that FRET has become a frequently used tool to analyze functional properties of proteins, thus also channels and receptors. Both approaches described above, VCF and PCF, can be used accordingly. Out of a plethora of results, the following examples are mentioned in chronological order to demonstrate the power of the method: Cha and coworkers used lanthanide-based resonance energy transfer to determine previously defined distances between Shaker potassium channel subunits and changes of them evoked by voltage [94]. Chanda and coworkers [95] used in Shaker channels the lipophilic dipicrylamine, that distributes on either side of the lipid bilayer depending on the membrane voltage, as FRET acceptor for donor molecules attached to defined positions, which finally provided evidence that the S4 segment does not translocate across the lipid bilayer, contradicting the paddle hypothesis for voltage-dependent gating [96]. Nanazashvili and coworkers performed lanthanide-based FRET experiments on single-Cys dimeric constructs of the mammalian renal inward rectifier channel and observed that a rigid body rotation of the large CTD around the permeation axis is correlated with opening of the so-called HBC hydrophobic gate [97].

Using transition metal ion FRET In spHCN channels, Dai and coworkers determined by combining PCF and molecular modeling differences in the structural rearrangements between activated and inactivated channels and discovered that removing cAMP produced a largely rigid-body rotation of the C-linker relative to the transmembrane domain [98].

Moreover, FRET can be used elegantly for studies at the single-molecule level (smFRET) (for review see, [99]. Finally, it should be noted that various pairs of fluorescent proteins, as e.g., CFP and YFP or Cerulean and Venus, are widely used for FRET experiments, mostly to determine distances at equilibrium, but also for kinetic analyses of activation, as e.g. for metabotropic glutamate receptors [100, 101].

##### P2X receptors

For the analysis of conformational changes in P2XRs, available FRET studies are rare. Fisher and coworkers did experiments on the activation of P2X2Rs and they labeled the C-termini with either CFP or YFP [102]. Their data provided some mechanistic insight into the channel machinery, suggesting that, upon activation also, the cytosolic domain of P2X2Rs undergoes a conformational change. The authors also claim that the results might be consistent with an expansion and/or a rotation within a domain and that their data provide a time-resolved measure of state-specific gating motions. They also make a proposal for how a cytosolic domain may control ion channel permeability.

Another FRET approach was performed on heteromerization of P2XRs by Schneider and coworkers [38]. These authors showed that subunits of P2X4Rs and P2X7Rs can form a heterotrimeric channel; however, unlike previous observations for P2X2 and P2X3, these heteromers did not lead to a novel functionally discriminable P2XR phenotype. A further study on P2X7Rs investigated that the mutant P2X7R-Gln460Arg per se is not compromised in its function. However, its co-expression with wt P2X7Rs impairs several parameters of the receptor function [103]. The fruitful experiences from such studies in other channels should encourage researchers in the field of P2X receptors to perform further approaches.

### Measuring ligand binding

#### General aspects

**Fig. 5:**
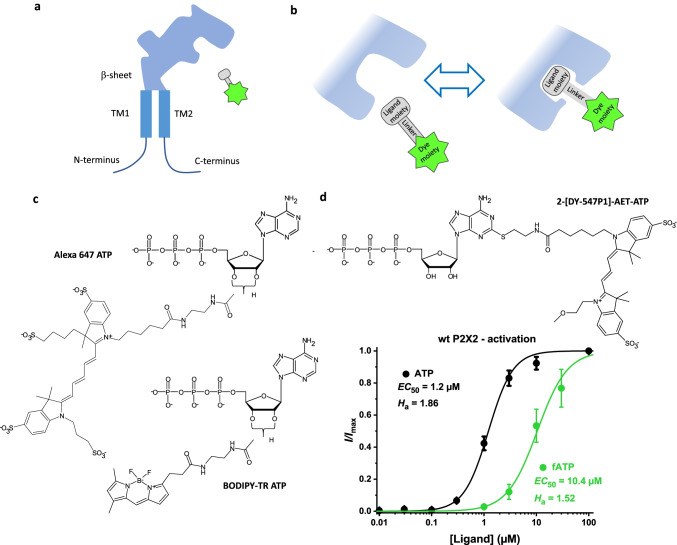
Fluorescence-labeled ATP derivatives (fATP) monitoring binding and conformational changes. **a** Cartoon of P2X subunit and fluorescent ligand. **b** Ligand-induced conformational change in the binding pocket. **c** Commercially available fATPs Alexa Fluor™ 647 2′-(or-3′)-O-(*N*-(2-aminoethyl) urethane adenosine-5′triphosphate (Bhargava 2013) and BODIPY TR 2′-(or-3′)-O-(*N*-(2-aminoethyl) urethan adenosine-5′triphosphate (Kowalski 2014). **d** Concentration-activation relationships with ATP and (2-[DY-547P1]-aminoethylthio) adenosine-5′-triphosphate on wt rP2X2 channels with the fitted EC50 value and Hill coefficient (from Sattler et. al. 2020)

The enormous triumph of fluorescence techniques in biosciences over the last two decades has brought up new and exciting possibilities to label also ligands and use them to stain receptors in the cell membrane. The construction principle is essentially simple: The natural ligand, either an agonist or a competitive antagonist, is used as the binding moiety to which a fluorescent dye is linked by an appropriate linker (Fig. 5). To mimic the function of a natural ligand, its fluorescent derivative should have ideally the same potency and efficacy, realizing thus the typical dynamic equilibrium between occupied and empty binding sites. It is therefore generally a good idea to keep the dye moieties small by using organic dyes, i.e., no antibodies, to preserve the high specificity of the ligand moiety for the desired receptor, i.e., to not chemically touch parts of the ligand molecule involved in the specific binding, to use sufficiently long linkers to keep the dye moieties away from all structures involved in the binding, and, finally, to use dyes with high brightness and photostability (c.f. Figure 5a, b).

Pioneering work in the field of labeled ligands was conducted for ligands of nicotinic acetylcholine receptors (nAchRs) already decades ago, using as fluorophores dansyl [104], *N*-(7-nitrobenz-2-oxa-1,3-diazol-4-yl) (NBD) [105], or pyrene [106], providing either agonists or antagonists with moderate affinity and low brightness. Five fluorescent compounds with improved properties were built by labeling the frog alkaloid epibatidine (EPB) [107], a potent agonist of various nAChRs, which was substituted by either coumarin, 6-chlorocoumarin, Cy3, Cy5, or Atto610. These compounds could be used to bind to and activate different nAChRs specifically. This means that, similar to the native ligand Ach, they reversibly bind and activate the receptors effectively, and thus mimic the properties of the natural ligand Ach successfully. The use of two further environment-sensitive residues further improved the usability of these fluorescent ligands [108]. On the other hand, the gating of the nAchRs is very fast whereas recording of the binding events requires sufficiently many photons. This discrepancy sets limits to the use of the mentioned fluorescent compounds for analyzing the activation gating of nAchRs.

Another class of ligand-gated ion channels has come into focus for studying ligand binding with fluorescent ligands; cyclic nucleotide-gated or modulated channels, comprising the family of voltage-independent cyclic nucleotide-gated (CNG) channels [109] and that of hyperpolarization-activated cyclic nucleotide-modulated (HCN) channels [110]. Notably, these channels are activated by the binding of cyclic nucleotides to a tetrameric cyclic nucleotide-binding domain at the channel inside. The search for fluorescent nucleotides with the specified features yielded that derivatives with an 8-substitution at the purine ring activate homo- and heteromeric olfactory CNG channels as well as HCN2 channels with similar potency and efficacy as do the respective natural cyclic nucleotides [111–114]. Subsequent systematic analyses quantified the influences of the used linkers and dyes [115, 116]. Using such fluorescent cyclic nucleotides, we developed the method of confocal patch-clamp fluorometry (cPCF), a technique that allowed us to gain from macropatches also orthogonal information, namely, about ligand binding in parallel to the ion currents, specifying ligand binding to homo- and heterotetrameric olfactory CNG channels [111, 117–119] and HCN channels [112]. Moreover, kinetics of binding and unbinding in parallel with activation and deactivation, respectively, could be used to substantiate rate constants in complex Markovian models describing the activation gating for both tetrameric channels [111, 113]. Recently, with the help of zero mode waveguides, one of these compounds has been used for single-molecule binding studies to the four cyclic nucleotide-binding domains in non-activated HCN2 channels [120, 121].

#### P2X receptors

With the knowledge that effective fluorescence-labeled agonists can indeed be built, it was intriguing that researchers tried to identify fluorescent ATP derivatives and use them to study the activation of P2X receptors. A first attempt was to use the fluorescent ATP derivative Alexa Fluor® 647 adenosine 5′triphosphate (Alexa-647-ATP) [122]. In this derivative, the dye is coupled to the 2′,3′ position of the ribose via an aminoethylcarbamoyl-linker (Fig. 5c). It was used to study rapid desensitization typical for P2X1Rs (see Fig. 1a). The half-maximum concentration for this process is as low as 3 nM which is much lower than the half-maximum concentration for activation, indicating the high affinity of the desensitized state. In another ATP derivative, BODIPY TR 2′-(or-3′)-O-(*N*-(2-aminoethyl) urethan adenosine-5′triphosphate (BODIPY-TR ATP), the dye was also coupled to the 2′,3′ position of the ribose (Fig. 5c). This derivative was used to investigate the agonist-induced movement of the ATP binding jaw, allowing the researchers to monitor the binding and gating for identifying amino acids not located in the binding pocket but influencing access to it [123]. It should be noted, however, that for none of these ATP derivatives, a concentration-binding relationship has been reported, not to mention a concentration-binding relationship in conjunction with channel activation.

**Fig. 6:**
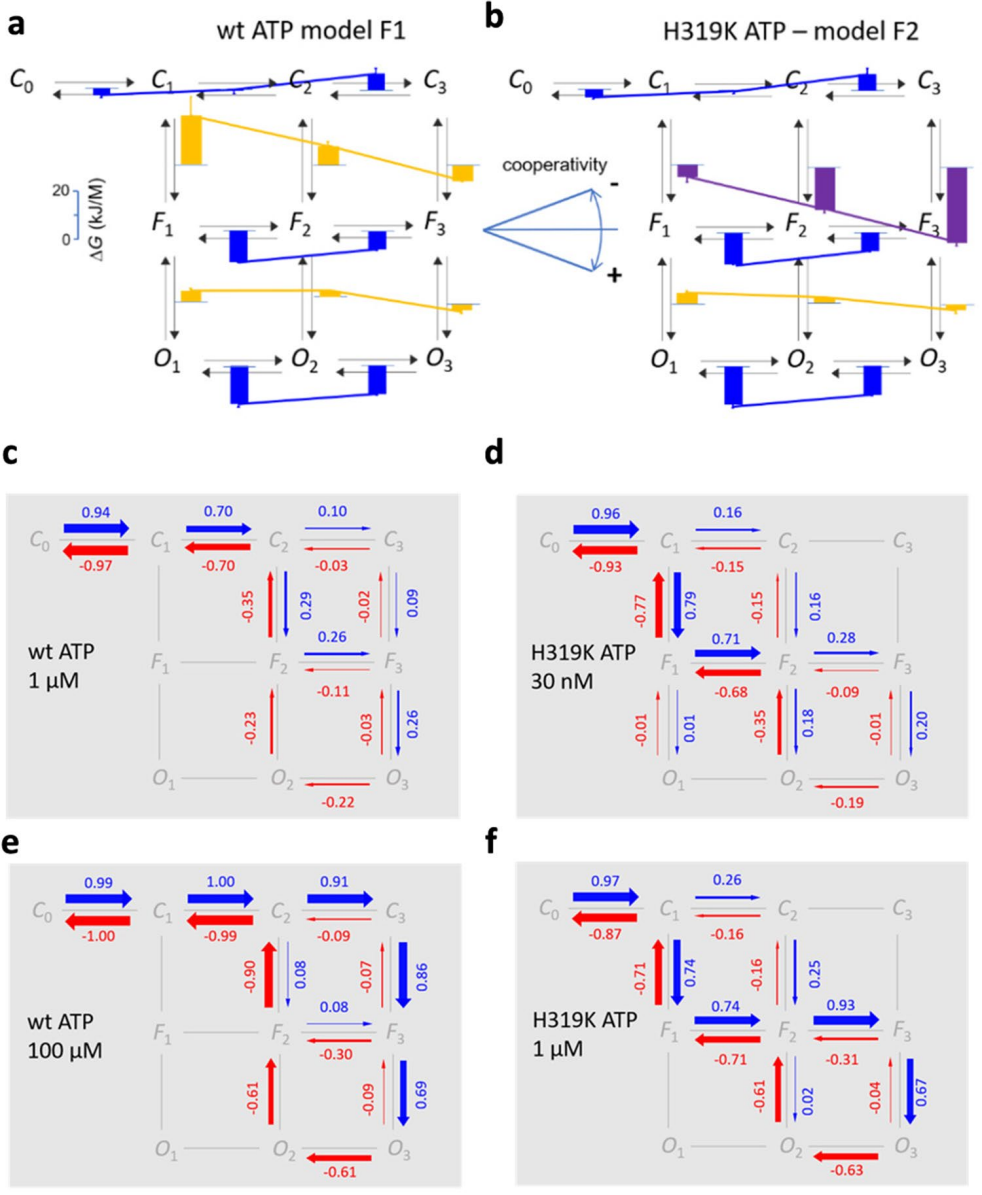
Energetics of the activation gating in P2X2 receptors. **a**, **b** Gibbs free energies for the association (blue bars) and isomerization (ochre and purple bars) in a model with three binding steps, flip reactions, and flipped-open isomerizations for wt P2X2 and sensitized mutant H319K channels. The blue lines indicate negative cooperativity for the binding steps. Ochre and purple lines indicate negative cooperativity for the isomerisations. **c–f** Probability fluxes when jumping to ATP (blue arrows) at the indicated concentrations and back to bath solution (red arrows). For details, see [14]

We recently followed another strategy to label ATP by a fluorophore and attached the dye DY-547P1 via an aminoethylthio-spacer (AET) to the 2 position of the purine ring, obtaining the compound 2-(2-[DY-547P1]-aminoethylthio) adenosine-5′-O-triphosphate (2-[DY-547P1]-AET-ATP or shortly fATP) (Fig. 5d) [124]. When using the mutant H319K of P2X2, showing a higher potency than wt P2X2 channels, fATP turned out to be a full agonist with respect to ATP. fATP reported to us the relative degree of binding by a bright fluorescence signal which allowed us to obtain the first concentration-binding relationship for a P2X receptor and, thus, the concentrations of half-maximum binding and half-maximum activation as well as the respective Hill coefficients. The Hill coefficients exceeded unity, even at an occupancy < 10%, suggesting cooperativity of the binding already for the first and second binding step. Nevertheless, the properties of the fATP were not perfect because the apparent affinity, i.e., the *EC*_50_ value, was by about one order of magnitude larger than that for ATP, suggesting relevant disturbance of the binding by either the linker or the dye moiety. The lower potency of ATP in wt P2X2Rs compared to H319K P2X2Rs was also observed with fATP. This hindered us technically to measure binding at the highest concentrations [124]. Despite the mentioned functional deficiencies of fATP, we could successfully use it together with natural ATP and the P2X2R mutant H319K for a global analysis strategy to unravel the gating in P2X2Rs [23]. In particular, we demonstrated that the steep concentration-activation relationship in wild-type channels is caused by a subunit flip reaction with strong positive cooperativity, overbalancing a pronounced negative cooperativity for the three ATP-binding steps (Fig. 6a, b). As a consequence, the net probability fluxes in the model generate a marked hysteresis in the activation-deactivation cycle (Fig. 6c–f). We also showed that the predicted fATP binding matches the binding measured by fluorescence. Hence, our analysis of orthogonal data generated essentially new insights into the intricate activation process of P2X2Rs.

Together, we speculate that, in the light of success of the systematic studies on the fluorescent ligands for CNG and HCN channels, further variation of both the dye and linker in fATP is very promising to obtain an optimized fATP fulfilling all desired features outlined above. This would provide then the opportunity to perform extended experiments with cPCF to further unravel the complex entanglement of ligand binding and activation, including the reciprocal functional relation between binding sites and channel pore (see Fig. 3) identified for HCN2 channels previously [112].

### Global fit analyses to analyze macroscopic currents and orthogonal signals

Global fit analyses of macroscopic ionic currents and, eventually, further orthogonal signals, by means of kinetic schemes are powerful to study the gating performance of ion channels. For analyzing multiple macroscopic currents solely, global fit analyses were used, e.g., to study the gating charge movement in Shaker channels [125], to quantify the intricate cooperativity of ligand binding in homotetrameric CNGA2 channels [114], to quantify voltage-induced activation and deactivation of homotetrameric HCN2 channels [126], and to identify the effects of permeating K^+^ ions on the *IV*-relationships of viral Kv channels [127]. Another strategy is to globally fit combined data from various mutations as shown for homotetrameric [128] and heterotetrameric CNGA2 channels [129].

As outlined above, significant improvement for the accuracy of the fits can be achieved when including orthogonal optical signals, as fluorescence signals from conformational changes or ligand binding, as shown for CNG and HCN channels [111, 113, 119]. For CNGA2 channels, this led to the identification of an intricate cooperativity for cGMP binding of “negative–positive-no” for the second, third, and fourth binding steps [111], whereas for HCN2 channels, this sequence was “positive–negative-positive” if the channels were pre-activated by hyperpolarizing voltage [113], whereas, in non-activated channels, this sequence was “negative-no-positive” [130].

We recently extended our global fit strategies to P2X2 receptors [23] and determined multiple rate constants when using four complex and intimately coupled kinetic schemes. The measurements were based on concentration pulses of either ATP or fATP, a fluorescent ATP derivative described above (see Fig. 5d), to either the wt P2X2R or its mutant H319K in order to elevate the apparent affinity. Moreover, we could describe the concentration-binding relationship for fATP with reasonable accuracy.

In the context of this review, we like to shortly summarize our strategy. We evoked concentration pulses to lifted HEK cells expressing the respective P2X2Rs. We than averaged the time courses of activation and deactivation of the corresponding currents when stepping to a defined ligand concentration and back to zero. These averaged time courses were transposed to open probabilities by using single-channel recordings. The resulting time-dependent open probabilities were subjected to the global fit strategy together with the respective concentration-activation relationships at equilibrium.

We specified a model with three closed states, three flipped states, and three open states (Fig. 6). Systems of first-order differential equations were used. Each differential equation describes the time-dependent change of the occupational probability of a considered state given by the sum of all inputs and outputs. A Matlab® program was written that treated the differential equations in a compact format in matrix notation to fit the data. The system of differential equations was solved numerically using the eigenvalue method. We obtained for each fit point of the diagrams numerical values of the occupation of states. The sum of the occupation of all open states is the open probability *P*_o_. In this way, the *P*_o_ vs. time courses and the *P*_o_ vs. concentration curves at equilibrium were calculated from the set of model parameters. To avoid different weight of the time courses and the equilibrium relationships, we introduced weighting factors, resulting in an equal number of effective fit points for all time courses and for all concentration-*P*_o_ relationships. From the rate constants, we obtained all equilibrium constants from which we computed free energies for the individual steps. Figure 6 a, b illustrate these energies and outlines also the effect of the mutation H319K. Hence, our analysis led us to the conclusion that the steep concentration-activation relationship in wt channels is caused by a subunit flip reaction with strong positive cooperativity, which overbalances a pronounced negative cooperativity for the three ATP binding steps [23].

These results allowed us further to compute the net probability fluxes in the specified model and to identify a marked hysteresis in the activation-deactivation cycle. We also identified the transition pathways [131–133] in our model for a given ligand step (Fig. 6c–f).

For activation, the predominant pathway to the most relevant open state *O*_3_ runs along *C*_1_–*C*_2_–*F*_2_–*F*_3_, of which *F*_2_ is less stable than the others. When applying the saturating concentration of 100 μM ATP, the transition pathway runs predominantly through the closed (non-flipped) states, and opening is exclusively generated by *O*_3_ via *F*_3_. The transition pathway for deactivation differs from the activation pathway notably. Whether activation was partial or complete, independently, it runs with high preference through the double-liganded states *O*_2_–*F*_2_–*C*_2_ to *C*_1_ and *C*_0_, generating a pronounced hysteresis. In H319K, the net probability fluxes differed substantially: at 30 nM ATP, again a concentration near the *EC*_50_ value, the binding of already the first ligand generates a significant flip *C*_1_–*F*_1_, and via *F*_2_ and *F*_3_, the relevant open states *O*_2_ and *O*_3_, respectively, are populated. In contrast to wt channels, the saturating concentration of 1 μM ATP also favors the pathway *C*_1_–*F*_1_–*F*_2_–*F*_3_ to generate predominant opening from the triple liganded flipped state *F*_3_. Notably, also at the equal ATP concentration of 1 μM, the net probability fluxes in wt and H319K channels differ upon activation due to the mutation.

Similar to wt channels, the predominant transition pathway for deactivation in H319K differs significantly from that of activation, in particular at the high ATP concentration of 1 μM, where it runs along *O*_3_–*O*_2_–*F*_2_ in contrast to activation which employs *F*_3_, generating again pronounced hysteresis for the closed-open isomerization. In contrast to wt, in H319K, the flipping runs predominantly in both activation and deactivation mainly without hysteresis along the pathway *C*_1_–*F*_1_–*F*_2_.

Our analyses suggest that it is indeed three ATPs that contribute to the activation gating of P2X2Rs, but also that only two or even one ATP can partly activate the channels. Hence, our global fit strategy reconciles earlier, apparently contradictory findings on the number of ligands required for channel opening (see above). Together, a global fit analysis of combined data sets, as described briefly here and reported recently [23], is a powerful tool to analyze also the gating of P2XRs, in case a plausible and appropriate model has been identified, which is always a daunting task.

## Outlook

Since cloning of the P2X receptors 25 years ago, remarkable progress has been made in understanding the relation between channel structure and function. The first high-resolution structures of closed and open states from zfP2X4 provided a framework for interpreting results from biochemical and biophysical experiments in more detail. Extended functional approaches of combined electrophysiological recordings and optical recordings, reporting either conformational changes or ligand binding, are very promising to further unravel the intricate gating of P2X receptors.

## Data Availability

All data are available upon request.
